# Assessment of small-intestine permeability in healthy Nigerian children is altered by urinary volume and voiding status

**DOI:** 10.1371/journal.pone.0253436

**Published:** 2021-09-20

**Authors:** Ibukun Afolami, Folake Olukemi Samuel, Martin Mwangi, Michael Oderinde, Marlies Diepeveen-de Bruin, Alida Melse-Boonstra

**Affiliations:** 1 Department of Human Nutrition, University of Ibadan, Ibadan, Nigeria; 2 Department of Human Nutrition, Wageningen University & Research, Wageningen, the Netherlands; Indiana University School of Medicine, UNITED STATES

## Abstract

**Objective:**

This study aimed to uncover the effect of voided urinary volume on small intestine permeability ratios in healthy children.

**Methods:**

We assessed small intestine permeability in 155 apparently healthy children, aged 3–5 years old, without any visible symptoms of disease, in a rural, malaria-endemic setting in Nigeria, using a multi-sugar test solution, comprising lactulose, sucrose, mannitol, and rhamnose. Children were categorized into low urinary volume (LV) and high urinary volume (HV), based on the volume of urine voided per kg body weight per hour. LV children voided less than 25^th^ percentile of the total population, while HV children voided greater than 75^th^ percentile of the total population. Urinary volume excreted over a 90-minute period after administration of the test solution was measured, and differences in sugar ratios were compared between children with high (HV) and low urinary volumes (LV), as well as between children who voided (VC) or who were not able to void (NVC) before administration of the test solution.

**Results:**

Urinary mannitol and rhamnose recovery were 44% (p = 0.002) and 77% (p<0.001) higher in HV children compared to LV children respectively, while urinary lactulose recovery was 34% lower (p = 0.071). There was no difference in urinary sucrose recovery between groups (p = 0.74). Lactulose-mannitol ratio, lactulose-rhamnose ratio and sucrose-rhamnose ratio were all significantly higher in children in the LV group compared to children in the HV group (p<0.001). In a multiple regression analysis, urinary volume and voiding status combined, explained 13%, 23% and 7% of the variation observed in lactulose-mannitol, lactulose-rhamnose and sucrose-rhamnose ratios, respectively.

**Conclusion:**

Sugar permeability ratios vary significantly with total urinary volume in multi-sugar small-intestine permeability tests. Voiding status before sugar administration appears to influence lactulose recovery, lactulose-rhamnose and sucrose-rhamnose ratios independently of total urinary volume. Evidence from this study suggests the need to take urinary volume into account when conducting multi-sugar small-intestine permeability tests.

## Introduction

Gut permeability is a terminology used to describe the control of material exchange between the lumen of the Gastro Intestinal Tract (GIT) and the rest of the internal body system. This control implies a physical barrier: comprising of the epithelial cell-lining of the gut, and a mucus layer; and a chemical barrier: comprising of digestive secretions, immune molecules, inflammatory mediators, and antimicrobial peptides [1]. Under certain conditions, such as during an infection or disease, the morphology and/or physiology of epithelial cells can be impaired, thus, leading to an abnormal permeability in the nutrient-absorbing region of the gut [2, 3]. This phenomenon is observed in certain enteropathies and microbial infections and is often used as an indicator of gut integrity [4–8].

Gut integrity can be assessed by measuring the transcellular and paracellular transport of certain high and low-molecular-weight sugars across the nutrient-absorbing regions of the gastro intestinal tract [7]. These transport systems have been shown to follow diverse mechanisms amongst which include passive paracellular diffusion, active influx transport, active efflux transport and transcytosis [2, 9, 10] Out of all the sugars studied, lactulose, rhamnose and mannitol have emerged to be the most common and most widely studied [11, 12]. Traditionally, gut permeability is expressed as a ratio of the fractional excretion of a larger sugar molecule (e.g. lactulose) to that of a smaller one (e.g. mannitol or rhamnose). A higher urinary lactulose concentration thus reflects a higher permeability (paracellular transport), whereas, a lower urinary mannitol or rhamnose concentration reflects an impaired cell surface area (transcellular transport) [13].

Gut permeability assessment, using the lactulose-mannitol or lactulose-rhamnose test, therefore makes it relatively easy to determine gut integrity in a non-invasive manner.

However, one major concern commonly reported using this test, is a high between-subject variation in sugar ratios [14, 15]. Some of these variations appear to be methodological [14, 16]. Multiple conditions ranging from fluid loading [17, 18] to severe diarrhoea can reduce the volume of urine voided during gut permeability tests. These may also contribute to the variations in permeability ratios and lead to errors in the interpretation of gut permeability.

We specifically aimed at assessing the effect of voiding status and urine volume on small intestine permeability ratios. Based on evidence that lactulose and mannitol sugars can already be found in the colon within two-hours of dosage [15]. For the purpose of the present study, we specifically tested small-intestine permeability in healthy children using a multi-sugar permeability test with a ninety-minute urine collection protocol to eliminate any possibility of absorption from other regions of the gut outside the small intestine. Specifically, our aim was to assess the effect of voiding status and urine volume on small-intestine permeability ratios.

## Methods

### Study design and population

This study was part of an 18-week Randomized Control Trial (RCT), conducted between December 2015 to April, 2016 in Telemu, Ilemowu and Asamu communities, in Osun State, south-west Nigeria. The primary aim of the RCT was to establish a proof-of-principle on the efficacy of pro-vitamin A biofortified (yellow) cassava on the total body vitamin A pool in children 3–5 years old. Recruited children were enrolled in a pre-school, established for the purpose of the study and were divided into two groups: an experimental group (n = 88 children), who fed on foods prepared with yellow cassava, and a control group (n = 88), who fed on foods prepared with white cassava. Study ethical approval was obtained by the Ethical Review Board of Wageningen University (the Netherlands), University of Ibadan (Nigeria), and Osun State Ministry of Health (Nigeria). Parental consent was obtained before the commencement of the study during family visits and community meetings. All parents signed an informed-consent before their children were eligible to participate in the study. The study is registered on www.clinicaltrial.gov under the identification No. NCT02627222.

### Anthropometric assessment

We measured participants’ weight and height using a weighing scale and stadiometer (SECA model No. 887 7021094, Germany). Children’s ages were verified by asking each mother/guardian to present the child’s birth certificate or immunization card. The computed ages for 75 children, were based on verbal recall from their parent or guardian. Height for age z-scores (HAZ), and weight-for-height z-scores (WHZ) were computed using WHO Anthro (version 3.2.2).

### Small intestine permeability tests

For the small-intestine permeability assessment, study participants were pooled from the two groups of the RCT. Twenty-one (n = 21) children were excluded because they were either absent from school or were not fasted. One hundred and fifty-five (n = 155) healthy children participated in the small intestine permeability test, which was conducted two weeks before the end of the RCT. All children were dewormed with a single dose (400mg) of albendazole and 300 mg prazinquantel at the beginning of the study, and were monitored for any form of sickness. Between the period of recruitment and small intestine permeability test, all the children who were diagnosed with malaria were treated, after which, they became eligible for the test. Participants were therefore considered healthy before the commencement of small intestine permeability test.

The test solution contained Lactulose (Sigma 61360; MM = 342.30 g/mol), sucrose (Sigma S9378; MM = 342.30 g/mol), D-Mannitol (Sigma M4125; MM = 182.17 g/mol), and L-rhamnose monohydrate (Sigma R3875; MM = 164.16 g/mol). Parents were reminded on the day before the test to ensure that their children came fasted. On the morning of the test, prescheduled children were assigned to small groups to ensure good supervision. Every child was carefully monitored by trained research assistants throughout the period of urine collection. Children were aided to void before sugar dosage, under supervision. Some children were unable to void, and these were therefore categorised separately during data analysis.

Forty grams each of lactulose and sucrose; and twenty grams each of mannitol and rhamnose, was weighed into a clean measuring beaker, and 1 litre of potable water was added. The solution was mixed by stirring with a clean spoon. After the dissolution of all the solids, the solution was aliquoted into 25-mL medicine cups. Each child was administered this 25 mL portion containing 1.0 g each of the high molecular weight sugars (lactulose and sucrose) and 0.5 g each of the low molecular weight sugars (rhamnose and mannitol). This was followed immediately by giving 100–200 mL potable water to induce urine production. Sugar solutions were prepared, at most, ten minutes before dosage. Urine was collected within 90 minutes after sugar dosage into labelled disposable plastic containers, and the exact time interval between sugar dosage and urine collection was noted. Two drops of 20% chlorohexidine was added to urine samples to prevent bacterial degradation of the sugars. Urine weight and specific gravity were measured immediately after collection, using a weighing scale (Kern & Sohn, D-72336, Germany) and urinalysis strip (Surescreen diagnostics, Derby, UK). Urinary volume was calculated by dividing the mass by the specific gravity. Sample aliquots (5 mL) were stored at -20°C between the time of collection and analysis.

Urinary lactulose, mannitol, sucrose and rhamnose concentrations were measured by gas-liquid chromatography, as described by Jansen *et al*. (1986) [19], using Tri-Sil-TBT derivatization, followed by separation on fused silica column, flame ionization detection and quantification by an internal standard method. All analyses were conducted in the laboratory of the Division of Human Nutrition and Health, Wageningen University and Research, the Netherlands. Internal controls were utilized according to the established laboratory protocol. Analysis runs which did not meet ±2 standard deviations of control mean were repeated until this requirement was met.

### Calculations and statistical analyses

The percentage recovery of each sugar in urine was calculated by multiplying the concentration (mg/ml) of the sugar in urine, by the total volume of urine collected over 90 minutes. Sugar recovery ratios were calculated by dividing the high molecular-weight sugars recovered in urine (i.e. lactulose or sucrose) by the low molecular weight sugars (i.e. mannitol or rhamnose). Data was entered using Stata version 13 (StataCorp, Texas, USA). To test for the effect of urinary volume on sugar recoveries, we created two contrasting categories of urinary volume, namely, low volume (LV) and high volume (HV). Low volume was defined as urinary volume equal to or lesser than the twenty-fifth percentile of the population sample, while high volume was defined as urinary volume equal to or greater than the seventy-fifth percentile of the same population sample. Children were also categorised into two groups: “voided children” (VC) and “non-voided children” (NVC) based on whether they voided or not, before administering the sugar solutions. Group mean comparisons were analysed using independent t-test statistics. A multivariate regression analysis was also conducted to determine the independent effect of urinary volume, voiding status, urine specific gravity, HAZ, WAZ and WHZ on lactulose/mannitol or lactulose/rhamnose ratio. We controlled for the confounding effect of age, gender and intervention group a posteriori, by including these variables as covariates in the regression model. Percentage recoveries for lactulose, sucrose, mannitol, as well as, lactulose-mannitol and lactulose-rhamnose ratios, were log-transformed for inferential statistical analyses. Geometric means, with their 95% confidence intervals are reported for all log-transformed variables, while arithmetic means are reported for non-transformed normally-distributed variables. All variables were tested for normality and skewness, using histogram and normality plots; and homoscedasticity, using the Levene’s test. Statistical analyses was conducted using Stata (version 13) software.

## Results

The mean age of all the children who participated in the assessment was 53.6 months; their average height-for-age z-score was -1.00, while their weight-for-height z-score was -0.64 (Table 1). No significant difference was observed in height-for-age (HAZ), and weight-for-height (WHZ) z-scores between the LV and HV groups (Table 1).

**Table 1:** Sugar recovery and recovery ratios in low and high urinary groups.

	Low Urinary Volume (LV)	High Urinary Volume (HV)	All groups 95% CI	Group Comparison	P-value
	(n = 39)	(n = 38)	(n = 155)		
Mannitol Recovery ^b^ (%)	3.62	5.19	4.76	1.44*	0.002
	[2.97, 4.39]	[4.58, 5.89]	[4.39, 5.15]	[1.14, 1.81]	
Rhamnose Recovery ^a^ (%)	1.66	2.94	2.46	1.28*	<0.001
	[1.39, 1.92]	[2.55, 3.32]	[2.28, 2.65]	[0.82, 1.74]	
Lactulose Recovery ^b^ (%)	0.114	0.090	0.097	1.34	0.071
	[0.92, 0.14]	[0.07, 0.11]	[0.087, 0.108]	[0.54, 1.03]	
Sucrose Recovery ^b^ (%)	0.049	0.046	0.050	1.06	0.74
	[0.036, 0.068]	[0.037, 0.058]	[0.044, 0.057]	[0.72, 1.58]	
Lactulose Mannitol Ratio ^b^	0.032	0.016	0.020	1.93*	<0.001
	[0.027, 0.037]	[0.013, 0.021]	[0.018, 0.023]	[1.43, 2.59]	
Lactulose Rhamnose Ratio ^b^	0.081	0.031	0.045	2.58*	<0.001
	[0.067, 0.098]	[0.024, 0.041]	[0.040, 0.051]	[1.86, 3.56]	
Sucrose Mannitol Ratio ^b^	0.014	0.009	0.010	1.51*	0.016
	[0.011, 0.017]	[0.007, 0.012]	[0.009, 0.012]	[1.08, 2.11]	
Sucrose Rhamnose Ratio ^b^	0.036	0.017	0.023	2.08*	<0.001
	[0.027, 0.046]	[0.013, 0.022]	[0.020, 0.027]	[1.45, 3.02]	
Age (months) ^a^	53.6	53.9	53.6	0.38	0.89
	[49.5, 57.6]	[50.1, 57.8]	[51.8, 55.3]	[-5.8, 5.1]	
HAZ ^a^	-0.90	-1.01	-1.00	0.12	0.69
	[-1.30, -0.50]	[-1.47, -0.56]	[-1.22, -0.77]	[-0.47, 0.71]	
WHZ ^a^	-0.68	-0.73	-0.64	0.05	0.80
	[-0.96, -0.40]	[-1.05, -0.40]	[-0.80, -0.48]	[-0.37, 0.47]	

^a^ Values are arithmetic means, (interquartile range) and group comparisons are expressed as arithmetic differences,

^b^ Values are geometric means (interquartile range), and group comparisons are expressed as arithmetic ratios i.e. LV/HV or HV/LV as the case may be

* Group difference is significant i.e. p<0.05.

### Sugar recoveries and small intestine permeability ratios in LV and HV groups

**Fig 1:**
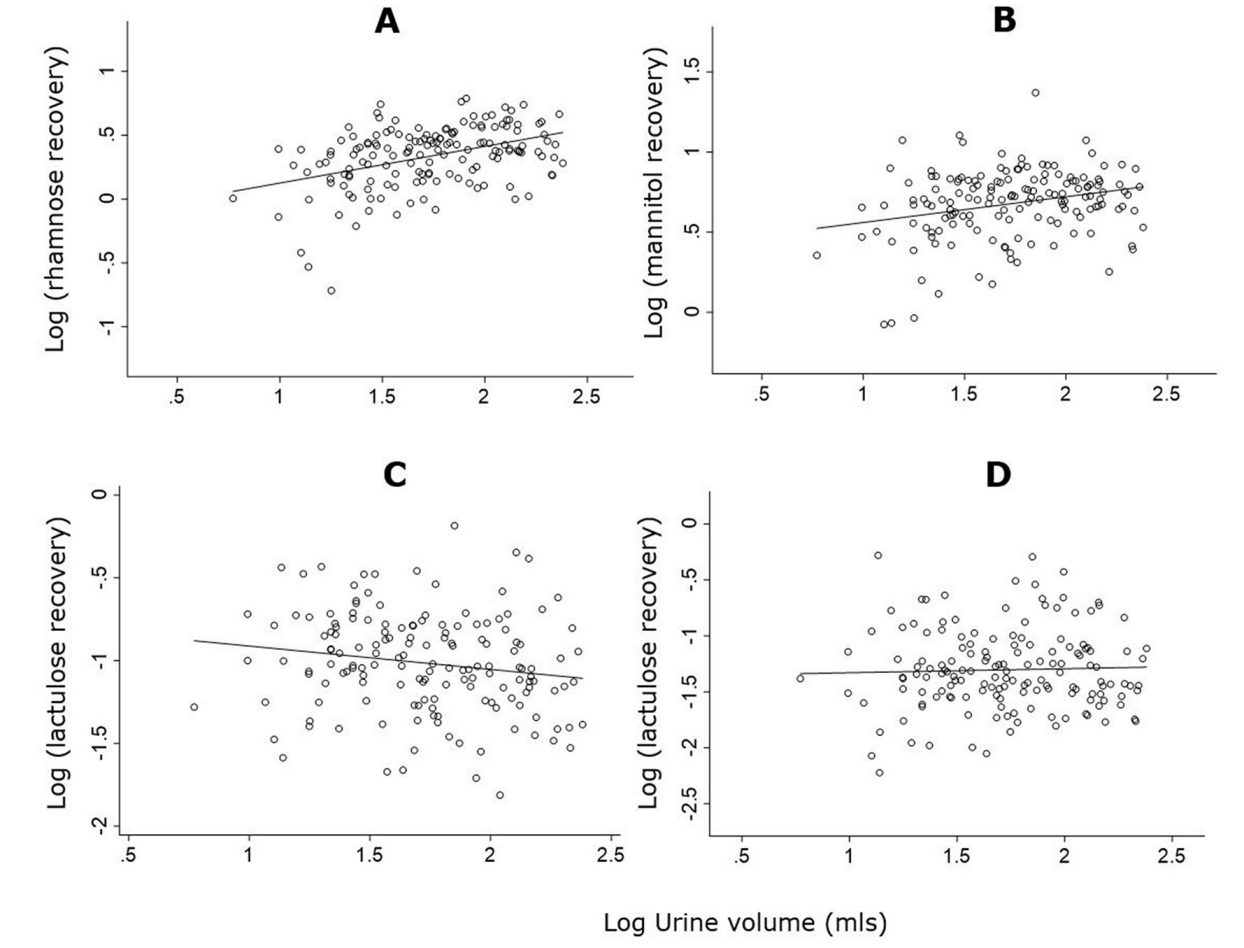
Correlations between sugar recoveries and urine volume. A = rhamnose recovery (r = 0.41 [p<0.001]); B = mannitol recovery (r = 0.26 [p<0.001]); C = lactulose recovery (r = -0.16 [p = 0.004]); D = sucrose recovery (r = 0.035 [p = 0.67]).

Children who voided 4.92 ml of urine per kg body weight or more (HV group, void > 75^th^ percentile), had higher recoveries of urinary mannitol (+44%, p = 0.002) and rhamnose (+77%, p<0.002) compared to children who voided 1.28 ml of urine per kg body weight or less (LV group, void> 25^th^ percentile). At the same time, children in the HV group had lower recoveries of lactulose (-34%, p = 0.071). Sucrose recovery was not statistically significant between groups (p = 0.74) (Table 1 and Fig 1).

**Fig 2:**
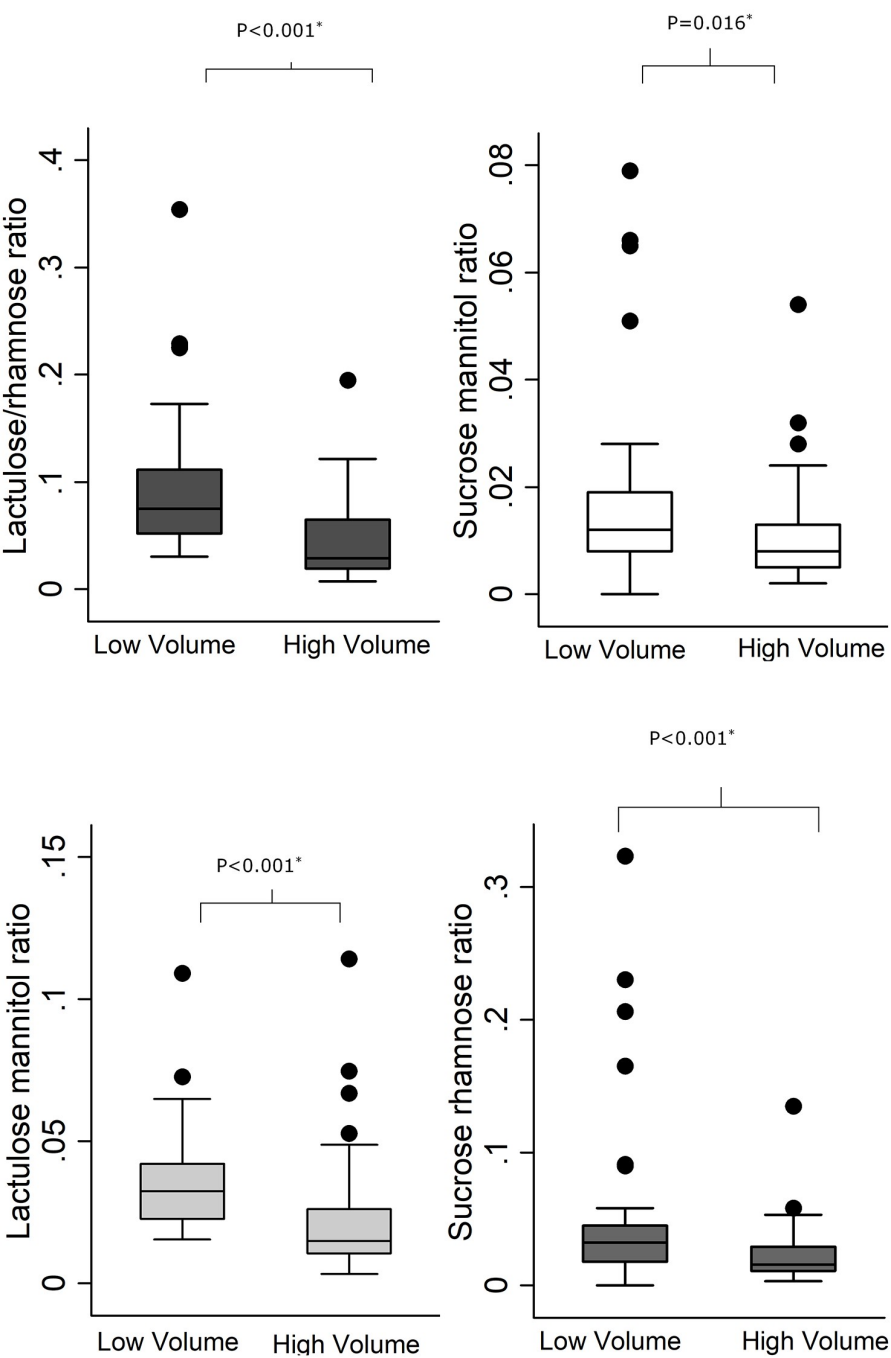
Sugar recovery ratios at low and high urinary volumes; 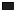 Lactulose rhamnose ratio; 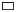 sucrose-mannitol ratio; 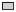 lactulose-mannitol ratio; 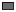 sucrose-rhamnose ratio.

Sugar recovery ratios were all significantly higher in the LV group, compared to the HV group: lactulose-mannitol ratio (p<0.001), lactulose rhamnose ratio (p<0.001), sucrose-mannitol ratio (p = 0.02), and sucrose-rhamnose ratio (p<0.001) (Table 1; and Fig 2).

### Comparison of non-voided (NVC) and voided (VC) groups

There was no differences in HAZ (p = 0.6), WAZ (p = 0.4), WHZ (p = 0.3), age (p = 0.07) and gender (p = 0.3) between the NVC and VC groups. Children in the NVC group (n = 22) voided a mean volume of 3.34ml/kg body weight/hour, equivalent to an average volume of 72.9 ± 54.2 ml of urine, whereas, children in the VC group (n = 122) voided mean volume of 3.28 ml/kg body weight/ hour, equivalent to an average volume of 72.4 ml ± 56.6 ml of urine (p = 0.6). However, children in the NVC group had a significantly higher urinary density (1.0165g/L vs 1.0134 g/L, p = 0.03). Furthermore, there was a 36% increment in lactulose recovery in the NVC group (p = 0.05).

Regression analysis indicated that urinary volume and voiding status of children explained 13%, 23% and 7% of the variation in lactulose-mannitol ratio, lactulose-rhamnose ratio and sucrose-rhamnose ratio respectively. Also, a 1% increase in urinary volume significantly corresponded to a 0.3%, 0.4% and 0.2% decrease in lactulose-mannitol ratio, lactulose-rhamnose ratio, and sucrose-rhamnose ratio respectively (Table 2). Age and gender variables did not make any significant contribution to the model, however, they were retained in the model. Similarly, urine specific gravity, HAZ, and WHZ did not also make any contribution to the model, hence, these were removed from the regression model.

**Table 2:** Multivariate linear regression analysis of the association between sugar recovery ratios and urine volume.

Regression	Log (urine volume)			Voiding status			
Model	X_1_			X_2_			
	β	SE(β)	P-value	β	SE(β)	P-value	R^2^ (adjusted)
Lactulose-mannitol ratio	-0.357	0.066	0.000	-0.098	0.063	0.123	0.131
Lactulose-rhamnose ratio	-0.487	0.071	0.000	-0.206	0.068	0.003 *	0.231
Sucrose-mannitol ratio	-0.127	0.081	0.121	-0.070	0.078	0.371	0.010
Sucrose-rhamnose ratio	-0.269	0.088	0.003	-0.177	0.084	0.037 *	0.075

Model variables were adjusted for age and gender;

* means model is significant, i.e. p<0.05.

## Discussion

In this study, we demonstrate that sugar recoveries and permeability ratios obtained during small intestine permeability assessments in relatively healthy children, are to a non-negligible extent, dependent on the volume of urine excreted during the sugar permeability test. In addition, the result of the test depends on whether the child was able to void before commencement of the test or not. This study therefore clearly suggests that the assessment of small intestinal permeability is dependent on volume of urine voided.

Our study reproduces similar findings reported previously in a five-hour gut permeability assessments for rhamnose recovery [3] and mannitol recovery [20], although we used a ninety-minute small intestine permeability assessment. In these studies, urinary volume was shown to contribute significantly to the variation in sugar permeability ratios. Rhamnose recovery was generally lower than mannitol in equal volumes of urine, despite similar doses of the two sugars were administered. This pattern has been reported in previous studies involving multi-sugar assessment, [21, 22], however, it is not very clear whether this is attributable to membrane transport mechanisms (i.e. active transport versus passive diffusion across gut membrane) or competition for membrane transporters by other sugar solutes present in the solution. Menzies et al, (1990) [21] observed that in a multi-sugar test, involving lactulose, L-rhamnose, and mannitol, mannitol reduced intestinal uptake of rhamnose and lactulose [21].

Furthermore, we observed a different relationship between voiding status and percentage recoveries for lactulose sugar, which appears to be independent of excreted urinary volume. Even though the total urinary volume produced by both voided and non-voided groups was not different, there was a 36% increment in lactulose recovery in the non-voided group. This could possibly reflect more concentrated urine due to a lower hydration state in this group, especially because the group also had a slightly higher urine specific gravity. However, this does not explain why only lactulose recovery was higher whereas recoveries of the other sugars did not differ from the group who voided before the test.

Our study does not answer the question why differences in urinary volume and voiding status result in differences in sugar recovery. Hence, it is not clear if LV and NVC truly had poorer gut function, or if the higher sugar recovery ratios rather result from other biologic conditions. Possible explanations are: 1) children with lower urinary volume or those who could not void before the test may have been in a poorer state of hydration; 2) differences in renal clearance and/or water resorption rate between children; 3) a combination of both. There were however no obvious differences in age, sex or malnutrition status between the LV and HV groups, nor between VC and NVC groups. They all lived in the same village and underwent the same screening procedure for eligibility to participate in the study. Since they were enrolled in an RCT, they largely ate the same food and had the same activity pattern. We did not assess hydration status of the children, although we did see a difference in urinary specific gravity between VC and NVC groups. It may be that children with poorer gut function coincidently were at the same time more often dehydrated, in which case their higher sugar recovery ratios truly reflect poorer gut function.

There is conflicting data in previous studies on the association between chronic and acute forms of malnutrition and gut permeability. Some studies have reported associations [23–26], while others show no association at all [27, 28]. In the present study, we found no association between small intestine permeability and height-for-age, weight-for-height and weight-for-age z-scores.

The mean values obtained for lactulose-mannitol and lactulose-rhamnose ratios from this study, compare very well with previous studies. Mean lactulose-mannitol, lactulose-rhamnose and sucrose-rhamnose ratios from this study were 0.020, 0.045 and 0.023 respectively, which falls within the range reported for healthy controls in non-hospital-based studies from African countries [29]. Additionally, we reviewed some relevant studies on gut permeability ratios conducted between 1979 and 2014 in Africa [23, 29–31], United Kingdom [32], Europe [3], South America [14, 33], Asia [6, 25, 28, 34, 35], and Australia [27]. In this, we made a distinction between hospital-based and non-hospital-based studies in order to differentiate gut permeability ratios reported in sick children from those reported in healthy children across different regions. Reported values for healthy children ranged from 0.030 to 0.054 for lactulose-mannitol ratio, and 0.015 to 0.070 for lactulose-rhamnose ratio. Brewster *et al*. (1997) [29] observed a higher range in African healthy children (lactulose rhamnose ratio, 0.034–0.096) compared to children in Europe (lactulose rhamnose ratio, 0.027). In this context, our results appear to fall somewhere at the lower limit, despite the fact that being a rural setting, infection rates were supposedly quite high amongst participants. A reasonable explanation for this is the deworming regimen provided at the beginning of the study.

An important limitation of the study was our inability to test the likely possibility that the total duration of urine collection influenced sugar recovery or gut permeability ratios [36]. However, it most likely would not have changed our results significantly, because the total volume of urine collected between the groups studied was almost the same. Also, we did not measure urine prior to dosing, which did not eliminate the possibility of dietary sources of the urine sugars. However, we expect that this possible source of bias was reduced by ensuring that children fasted before urine collection. In addition, the LC-MS MS method has been shown to be a preferrable method for measuring sugar concentrations in urine [13]. However, based on the low coefficient of variation of the laboratory controls used for rhamnose (2.2%), lactulose (7.7%) and sucrose (7.1%), we expect that any possible error in measurement was reduced to the bare minimum.

In conclusion, data from this study strongly suggests that diuretic conditions in children is a significant factor contributing to variations in small intestine permeability ratios, using sugar probes. We therefore recommend that urinary volume and voiding status be carefully monitored during gut permeability tests and future studies should compare urines sample parameters at baseline and after administering sugar tests.

## Supporting information

S1 Data Gut permeability data. (XLSX)
